# Effect of peripheral field loss on gait performance: a systematic review and meta-analysis

**DOI:** 10.3389/fnins.2025.1612793

**Published:** 2025-06-13

**Authors:** Shuwen Jia, Umar Muhammad Bello, Mei Zhao, Anqi Lyu, Ginny H. T. Wong, Benjamin Thompson, Allen Ming Yan Cheong

**Affiliations:** ^1^School of Optometry, The Hong Kong Polytechnic University, Kowloon, Hong Kong SAR, China; ^2^InnoHK, Centre for Eye and Vision Research Limited, Hong Kong Science Park, Hong Kong, Hong Kong SAR, China; ^3^Department of Physiotherapy and Paramedicine, School of Health and Life Sciences, Glasgow Caledonian University, Glasgow, United Kingdom; ^4^School of Optometry and Vision Science, University of Waterloo, Waterloo, ON, Canada; ^5^Research Centre for SHARP Vision, The Hong Kong Polytechnic University, Kowloon, Hong Kong SAR, China

**Keywords:** mobility, walking speed, gait performance, safety, visual field, peripheral vision loss

## Abstract

**Background:**

The peripheral visual field provides essential environmental information for safe locomotion. Deficits in peripheral field can adversely affect gait performance and safety. This review aimed to consolidate current knowledge on the impact of peripheral field loss on gait and to identify the key parameters for gait assessment.

**Methods:**

A comprehensive systematic search was conducted across AMED, CINAHL, PubMed, Scopus, and Web of Science databases, supplemented by a manual search on Google Scholar, covering the period up to November 2023. Eligible studies examining the relationship between peripheral field loss and gait performance were summarized and methodologically assessed using the Strengthening the Reporting of Observational Studies in Epidemiology (STROBE) quality rating tool. Meta-analysis was conducted using the Comprehensive Meta-analysis (CMA) software.

**Results:**

The review included 23 studies with a total of 3,085 participants. The average STROBE score was 19, ranging from 15 to 21. Walking speed was the most frequently assessed gait parameter, with peripheral field loss significantly associated with reduced walking speed (r = 0.40, *p* < 0.001). In addition, peripheral field loss correlated with an increased number of collisions, indicating compromised mobility safety. Moreover, alterations in spatiotemporal gait parameters, such as stride length and cadence, were also linked to peripheral field loss.

**Conclusion:**

Peripheral field loss is significantly associated with reduced walking speeds, altered gait characteristics, and impaired mobility safety during locomotion. Future research should adopt a standardized set of gait and mobility metrics to enhance cross-study comparisons among diverse patient populations.

**Systematic Review Registration:**

CRD42022297071.

## Introduction

Gait patterns and postural control are fundamental components of balance, essential for participating in daily activities (Gill et al., 1995). Variability in gait, often due to aging or disease, not only increases the risk of falls but also decreases the health-related quality of life (Shin et al., 2021; Ayoubi et al., 2015; Herssens et al., 2018; Brognara et al., 2019; Park and Kim, 2019; Montana and Bhorade, 2018; Ramulu et al., 2019). Alongside the neuromuscular system that directly regulates postural stability (Rueangsirarak et al., 2018; Khan and Andersen, 2021), our visual system provides crucial information about self-position (Black and Wood, 2005) and the surrounding environment (Patla, 1998), vital for safe locomotion (Rogge et al., 2021).

The relationship between gait performance and visual functions has been extensively investigated. Individuals with reduced vision typically walk slower than their aged-matched peers with normal vision (Wood et al., 2009; Timmis and Pardhan, 2012; Varadaraj et al., 2017). The peripheral visual field, in particular, provides essential environmental cues, such as locating obstacles, monitoring changes in ground terrain, and facilitating real-time feedback for step adjustments (Peli et al., 2016; Marigold, 2008). Therefore, even when visual acuity is relatively good, patients with restricted visual fields are more likely to miss hazards and adopt cautious gait patterns (De Alencar Gomes et al., 2018; Lee et al., 2021; Miller et al., 2018) This lack of environmental awareness can significantly reduce their physical activity levels, and the resulting decline in exercise may further worsen gait abnormalities (Lee et al., 2019).

Gait analysis is commonly used to assess mobility in various populations, including older adults and those with limited mobility such as stroke and Parkinson’s disease. Although there have been reviews on the effects of aging and diseases on gait (e.g., Parkinson’s disease; Fukuchi et al., 2019; Skiadopoulos et al., 2020; Nascimento et al., 2020; Zanardi et al., 2021), there is a scarcity of systematic reviews focusing on the impact of peripheral field loss (PFL) on gait variability (Geruschat et al., 1998; Friedman et al., 2007; Medeiros, 2021). This is an important knowledge gap, given that over 76 million individuals worldwide have restricted visual fields (Tham et al., 2014), with prevalence increasing with age (Zhang et al., 2021). The objectives of this review were to: (1) identify gait parameters used to study mobility in individuals with PFL; (2) assess the immediate and long-term effects of PFL on gait parameters; and (3) recommend appropriate and sensitive gait parameters for future research examining the effect of PFL on gait performance and the effectiveness of interventions designed to mitigate this impact.

## Methods

The methodology and reporting of the findings in this review follows the standards set by the Preferred Reporting Items for Systematic Reviews and Meta-Analyses 2020 (PRISMA-2020) guidelines (Page et al., 2021) The review protocol was registered with the International Prospective Register of Systematic Reviews (PROSPERO; Ref. No: CRD42022297071) in December 2021 before data extraction processes began. We adopted the PICO (Participants, Intervention, Comparators and Outcome) format to generate research questions (Higgins et al., 2019) The included studies involved participants with only PFL, characterized by normal visual acuity but restricted visual field due to ocular diseases. Simulated visual field loss was excluded, as participants typically do not have the opportunity to acclimate to the loss of peripheral vision and develop compensatory strategies. An intervention was not required, and baseline data were collated for intervention studies. The comparators and outcomes were healthy controls and kinematic/spatiotemporal gait parameters, respectively. The study conceptualization and development of the review protocol were undertaken by authors (SWJ, UMB, BT, and AMYC).

### Search strategy

The search terms were grouped under two themes, namely: ‘peripheral visual field loss’ and ‘kinematic and spatiotemporal gait parameters’. The theme ‘peripheral visual field loss’ included terms such as ‘visual field defect’ or ‘glaucoma’ to specify participants’ vision condition. The theme ‘kinematic and spatiotemporal gait parameters’ included terms related to walking, such as ‘gait speed’ and ‘mobility’. The electronic search involved combining terms under each theme using the Boolean operator ‘OR’. The search themes were combined using the Boolean ‘AND’ (Appendices 1 and 2 present the details of the search themes/terms and search strategy adopted for the CIHANL database, respectively). Articles written in Chinese and English were included because the study team included native speakers of both languages. Citation management software (EndNote X9, Clarivate Analytics, Philadelphia, Pennsylvania, USA) was used to organize the electronic search results and for deduplications. Two authors (SWJ and UMB) independently conducted the electronic search. Any discrepancies were resolved by consulting a third author (AMYC). A thorough manual search, including reference lists of the identified studies and forward references search using Google Scholar, was conducted to ensure no eligible studies were omitted. A secondary search was conducted on 30^th^ Nov 2023, covering the period from Dec 2021 to Nov 2023, to identify new literature added since the initial database search.

### Study eligibility criteria

Studies were included if they (1) assessed kinematic and/or spatiotemporal gait parameters among people with PFL caused by ocular disorders (e.g., glaucoma or retinitis pigmentosa) with or without an age-matched healthy control group or any intervention; (2) were cross-sectional or longitudinal/follow-up studies; and (3) were available in full text. Excluded studies were (1) review protocols; (2) systematic reviews; (3) conference abstracts; and (4) studies involving patients with PFL caused by neurological disorders, such as stroke as these may directly affect gait parameters (Khan and Andersen, 2021).

### Article screening

The identified studies via electronic search processes were sequentially screened at the title, abstract, and full-text phases by three of the authors (title and abstract screening: SWJ and UMB; full-text screening: SWJ and AQL). Any discrepancies identified by them during the screening phases were resolved by either discussion or consulting a third author (AMYC).

### Data extraction

The primary data for this study was the outcome of the gait parameters assessed in the included studies. Other relevant data extracted included study reference, study design, participants’ characteristics, baseline visual assessments, methods of gait assessment, and major results of the study. Data extraction was undertaken independently by SWJ and MZ using an extraction tool designed in Microsoft Excel. Disagreements between the authors during the data extraction process were resolved by discussion or by consulting a third author (UMB).

### Critical appraisal of the included studies

Quality appraisal of the included studies was performed using the Strengthening the Reporting of Observational Studies in Epidemiology (STROBE) checklist (Vandenbroucke et al., 2007). Although STROBE was not primarily designed for this purpose, because of the lack of validated tools to assess the quality of observational studies, systematic reviews and meta-analyses commonly used STROBE as a quality assessment tool (da Costa et al., 2011). The STROBE statement consists of 22 items scored using a ‘yes’, ‘no’, or ‘unclear’ ratings (Beckwée et al., 2017). It was developed to examine the strengths and limitations of observational studies included in systematic reviews for sound application of the study outcomes (Beckwée et al., 2017). The sum number of items scored as ‘yes’ in the checklist indicates the methodological quality of the study. The quality appraisal of the included studies was conducted independently by two authors (AQL and GW). Disagreements between the authors during the quality appraisal were resolved by further discussions or by consulting a third author (UMB).

### Data synthesis and statistical analysis

Authors UMB and SWJ synthesized the extracted data. We first synthesized the findings narratively due to study diversity. We conducted a narrative synthesis based on the study design and in line with the guidelines provided by the Centre for Reviews and Dissemination (Popay et al., 2006), encompassing quantitative analyses of the effect of PFL on gait parameters. Gait parameters from studies that recruited participants with PFL and age-matched healthy counterparts, with adequate outcomes were pooled for meta-analysis. We conducted meta-analyses using the Comprehensive Meta-analysis software (CMA version 4.0, Biostat Inc., Englewood, New Jersey, USA). We utilized the bias-adjusted standardized mean difference (Hedges’s g) and the correlation coefficient (r) as effect sizes, applying a random-effects model to account for variability among studies. The level of statistical significance for meta-analysis was set at *p* < 0.05. All numerical data were extracted from text, in-text tables, or supplementary material whenever possible.

## Results

**Figure 1:**
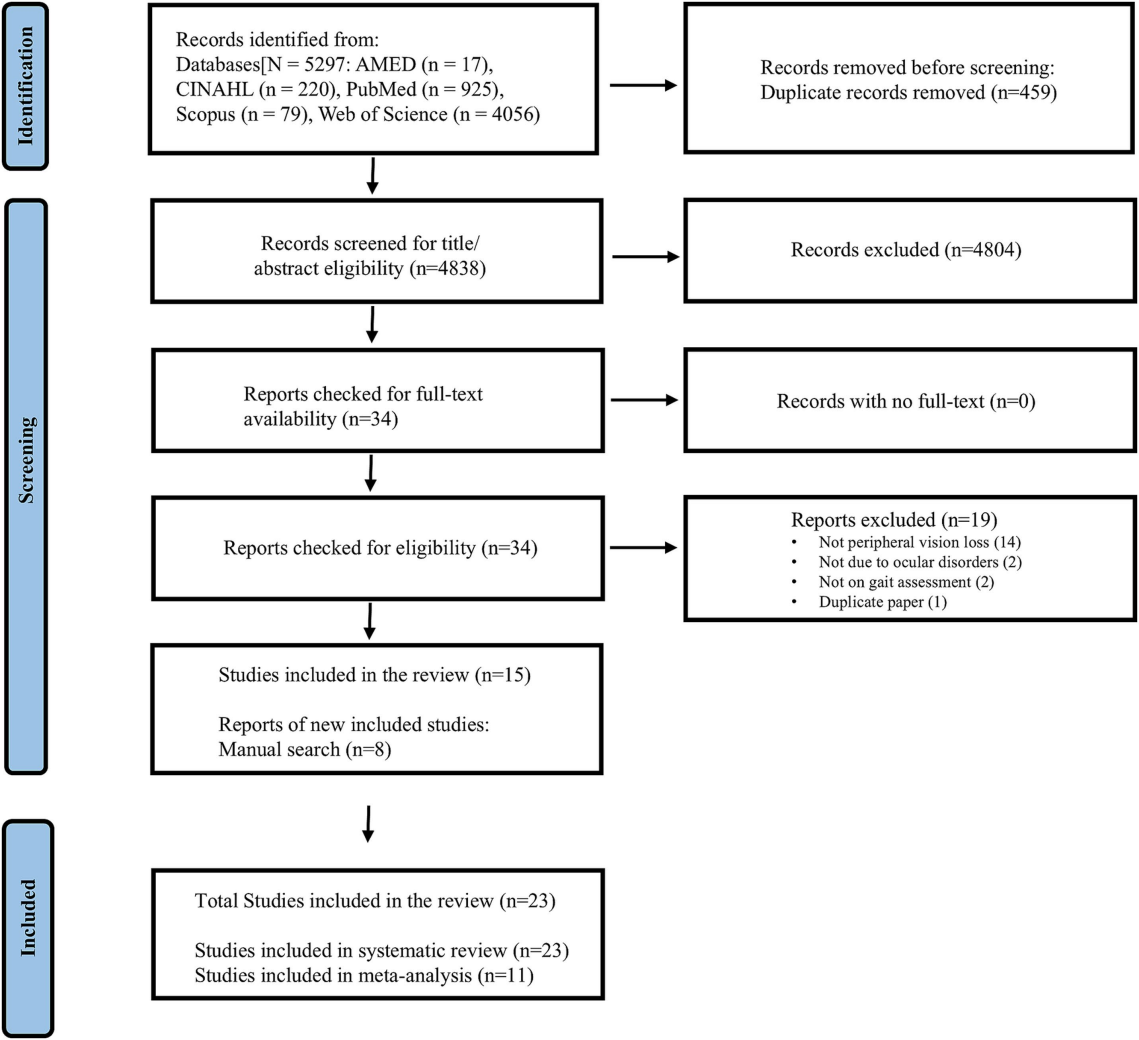
Study flowchart.

The electronic database search initially identified 5,297 studies. After removing 459 duplicates, we screened the remaining studies based on their titles, abstracts, and full texts. This process resulted in the identification of 15 studies that met the review criteria. Additionally, a manual search uncovered 8 more studies that were either previously overlooked or published after the initial search, bringing the total number of included studies to 23. Out of these 23 studies, 11 were eligible for inclusion in the meta-analysis with their characteristics summarized in Table 1. The reasons for excluding certain studies from the meta-analysis are summarized in Appendix 3. A flowchart detailing the search and screening processes is presented in Figure 1.

**Table 1:** Characteristics of the included studies in the meta-analysis.

Study reference	Participant characteristics	Visual Field	Gait assessments
Bertaud et al. (2021) France	Glaucoma *N* = 22 Age: 56.4 ± 10.1 F/M: 9/13 Normal *N* = 12 Age: 56.7 ± 10.1 F/M: 5/7	VF: HFA SITA standard 24–2 (IVF), Esterman IVF: Glaucoma: 32.3 ± 22.3 Normal: - Esterman score: Glaucoma: 91.2 ± 27.3 Normal: 116.1 ± 4.1	Walked at their preferred walking speed following an established route with obstacles (8 m)
Black et al. (1997) Australia	RP *N* = 10 Age: 45.2 ± 11 F/M: 6/4 Normal *N* = 9 Age: 46.8 ± 14 F/M: 7/2	VF: HFA binocular 30–1 (average visual field extent, degree) RP: 13.4 ± 5 Normal: >30	Walked at normal walking speed for 20 m with obstacles. (without cane)
Finger et al. (2016) Australia	Legal blindness (80% of patients were RP) *N* = 40 Age: 53 ± 16 F/M: 19/21	VF: Goldmann kinetic perimetry (binocular, % of VF remained) 11.8 ± 20.4	Walked at normal walking speed for 27 m with obstacles. (without cane)
Freitag et al. (2023) Germany	Glaucoma *N* = 19 Age: 70.7 ± 5.9 F/M: 10/9 Normal *N* = 30 Age: 70.9 ± 5.1 F/M: 17/13	VF (MD, Median | Range): Glaucoma OD: −0.67 | 25.97 OS: −1.09 | 22.00 Normal OD: 0.59 | 5.09 OS: 0.1 5 | 5.92	Participants walk normally forth and back over a 10-m track.
Geruschat et al. (1998) USA	RP *N* = 22 Age: 44.4 F/M: / Normal *N* = 16 Age: 38.2 F/M: /	VF Goldmann perimeter (Monocular, total area of functional retina in log unit) RP: OD: 2.29 mm^2^ (0 to 2.86 mm^2^) OS: 2.27 mm^2^ (1.0 to 2.82 mm^2^) Normal: mean of 2.82 mm^2^	Walked quickly following an established route with obstacles (49 m)
De Alencar Gomes et al. (2018) Brazil	Glaucoma *N* = 33 Age: 68.4 ± 8.0 F/M: 22/11 Normal *N* = 34 Age: 69.3 ± 7.9 F/M: 27/7	VF Octopus 1–2-3 (MD) Glaucoma: worse eye: −6.3 ± 3.7 better eye: −4.8 ± 1.8 Normal: -	Walked at a normal pace without obstacles (5.74 m)
Haymes et al. (1996) Australia	RP *N* = 18 Age: 44 (17–75) F/M: 10/8	VF Goldmann perimeter, (binocular kinetic visual, % of total VF) 4 to 89%	Walked along different routes at a comfortable pace (238 m, flat, unobstructed)
Lee et al. (2021) Korea	Glaucoma *N* = 15 Age: 72.87 ± 3.38 F/M: 11 /4 Normal *N* = 15 Age: 72.73 ± 3.88 F/M: 11/4	VF -	Walked without obstacles at a normal pace (6 m)
Miller et al. (2018) Canada	Glaucoma *N* = 20 Age74.3 ± 6.3 F/M: 14/6 Normal *N* = 20 Age: 70.7 ± 6.8 F/M: 14/6	VF HFA standard 30–2 (better eye, MD) −8.88 ± 5.57 (glaucoma) 1.10 ± 1.70 (normal)	Walked at normal speed and stepped to the center of a series of four sequential targets
Odden et al. (2020) USA	Glaucoma *N* = 231 Age (IQR): 70.6 (54, 75) F/M: 114/117	VF HFA standard 24–2,	Walked at normal speed without obstacles (4.88 m)
Turano et al. (1999) USA	Glaucoma *N* = 47 Age: 65.1 F/M:/ Normal *N* = 47 Age: 60.2 F/M:/	VF HFA SITA 24–2 (MD) Glaucoma: better eye: −10.1 ± 8.41 worse eye: −16.5 ± 10.00 Normal: - Esterman Glaucoma: Median: 87 Normal: Median: 94	Walked at a self-selected speed without obstacles. (29 m)

HFA, Humphrey Field Analyzer; IVF, Integrated visual field; MD, mean deviation; N, Number; F, Female; M, Male; RP, retinitis pigmentosa; SITA, Swedish Interactive Thresholding Algorithm; VF, visual field.

### Characteristics of included studies and participants

Among the 23 included studies, a total of 3,085 participants were recruited, with mean ages ranging from 17(Haymes et al., 1996) to 86(De Alencar Gomes et al., 2018) years. More females (*n* = 1,593) than males (*n* = 1,325) were recruited, although three studies did not report the sex distributions of their participants (Geruschat et al., 1998; Ivanov et al., 2016; Turano et al., 1999). The primary causes of PFL were glaucoma and retinitis pigmentosa. Twenty studies adopted a cross-sectional study design (Bicket et al., 2020; Black et al., 1997; Finger et al., 2016; Friedman et al., 2007; Geruschat et al., 1998; De Alencar Gomes et al., 2018; Hall and Barnes, 2011; Haymes et al., 1996; Lee et al., 2021; Ma et al., 2016a; Ma et al., 2016b; Mihailovic et al., 2020; Mihailovic et al., 2017; Miller et al., 2018; Odden et al., 2020; Turano et al., 1999; Bertaud et al., 2021; Lombardi et al., 2018; Shakarchi et al., 2019; Freitag et al., 2023), one used a longitudinal study design (Jian-Yu et al., 2021), and two utilized a quasi-experimental/randomized control trial design (Gunn et al., 2019; Ivanov et al., 2016). Detailed participants’ characteristics are presented in Appendix 4.

### Quality appraisal of the included studies

The methodological quality of the included studies is illustrated in Table 2. The average STROBE score was 19 out of 22, with scores ranging from 15(Ma et al., 2016a; Ma et al., 2016b)to 21(Jian-Yu et al., 2021; Mihailovic et al., 2020; Mihailovic et al., 2017; Miller et al., 2018; Odden et al., 2020). Higher scores indicate greater adherence to STROBE guidelines. Common limitations included insufficient details on sample size calculations (Bicket et al., 2020; Black et al., 1997; Finger et al., 2016; Friedman et al., 2007; Geruschat et al., 1998; Gunn et al., 2019; Hall and Barnes, 2011; Lee et al., 2021; Haymes et al., 1996; Ivanov et al., 2016; Jian-Yu et al., 2021; Ma et al., 2016a; Ma et al., 2016b; Mihailovic et al., 2020; Mihailovic et al., 2017; Odden et al., 2020; Turano et al., 1999; Bertaud et al., 2021; Lombardi et al., 2018; Shakarchi et al., 2019) limited descriptions of experimental settings (Black et al., 1997; Finger et al., 2016; Geruschat et al., 1998; De Alencar Gomes et al., 2018; Gunn et al., 2019; Hall and Barnes, 2011; Haymes et al., 1996; Ivanov et al., 2016; Ma et al., 2016a; Ma et al., 2016b; Miller et al., 2018; Turano et al., 1999), and inadequate disclosure of funding sources and their roles (Black et al., 1997; Friedman et al., 2007; Geruschat et al., 1998; De Alencar Gomes et al., 2018; Lee et al., 2021; Ma et al., 2016a; Ma et al., 2016b; Bertaud et al., 2021; Lombardi et al., 2018).

**Table 2:** Methodological quality assessment using STROBE.

	Item No	Bertaud et al. (2021)	Bicket et al. (2020)	Black et al. (1997)	Finger et al. (2016)	Freitag et al. (2023)	Friedman et al. (2007)	Geruschat et al. (1998)	De Alencar Gomes et al. (2018)	Gunn et al. (2019)	Hall and Barnes (2011)	Haymes et al. (1996)	Ivanov et al. (2016)	Jian-Yu et al. (2021)	Lee et al. (2021)	Lombardi et al. (2018)	Ma et al. (2016a)	Ma et al. (2016b)	Mihailovic et al. (2017)	Mihailovic et al. (2020)	Miller et al. (2018)	Odden et al. (2020)	Shakarchi et al. (2019)	Turano et al. (1999)	Total
Title& abstract	1																								23
Introduction																									
Background	2																								22
Objectives	3																								20
Methods																									
Study design	4																								18
Setting	5																								11
Participants	6																								22
Variables	7																								17
Data sources/Measurement	8																								22
Bias	9																								20
Study size	10																								3
Quantitative variables	11																								20
Stat. methods	12																								16
Results																									
Participants	13																								23
Descriptive data	14																								22
Outcome data	15																								23
Main results	16																								23
Other analyses	17																								23
Discussion																									
Key results	18																								22
Limitations	19																								20
Interpretation	20																								23
Generalizability	21																								23
Other information																									
Funding	22																								14
Item checked		17	20	18	18	19	19	17	19	20	18	16	20	21	20	17	15	15	21	21	21	21	20	17	
	Marked as “Yes” on STROBE-checklist																								
	Marked as “No” on STROBE-checklist																								
	Marked as “Unclear” on STROBE-checklist																								

### Narrative synthesis on the effect of peripheral field loss on gait parameters

Various methods were used to assess gait, ranging from basic timers to advanced devices like electric walking pathways and 3D cameras. Commonly examined parameters included walking speed (or percentage of preferred walking speed), errors (in terms of collision frequency), and spatiotemporal metrics (e.g., cadence, stride length). The influence of environmental challenges, and cognitive tasks were also examined.

#### Walking speed

Walking speed was the most frequently examined parameter, included in 19 studies. Ten investigations found that greater visual field loss was associated with reduced walking speed (Bicket et al., 2020; Finger et al., 2016; Haymes et al., 1996; Jian-Yu et al., 2021; Mihailovic et al., 2017; Turano et al., 1999; Friedman et al., 2007; Odden et al., 2020; Lombardi et al., 2018; Shakarchi et al., 2019) Eight cohort studies showed that participants with PFL walked more slowly than age-matched healthy controls (Black et al., 1997; Geruschat et al., 1998; Ivanov et al., 2016; Turano et al., 1999; Lee et al., 2021; Miller et al., 2018; Bertaud et al., 2021; Freitag et al., 2023). Although one study reported that glaucoma patients walked slightly faster than controls (De Alencar Gomes et al., 2018), the difference was clinically negligible. Factors affecting walking patterns such as lighting, walking course complexity, and dual-task conditions are summarized in Appendix 4. In general, low illuminance (e.g., below 101 lux) significantly reduced walking speed in all participants (Bertaud et al., 2021; Bicket et al., 2020; Black et al., 1997; Geruschat et al., 1998). Two studies (Bertaud et al., 2021; Black et al., 1997) examined the changes in walking speed under different lighting conditions and reported a greater reduction in walking speed in dim environments in people with PFL than those with normal vision. Complex walking course (Finger et al., 2016; Haymes et al., 1996; Turano et al., 1999) (e.g., with obstacles or variable path designs) and dual-task conditions (Miller et al., 2018; Gunn et al., 2019; Freitag et al., 2023) (e.g., additional counting or a visual search task) also slowed walking speed in participants with PFL and normal vision. Overall, most studies indicated that PFL was associated with or caused reduced walking speed, especially under challenging walking conditions.

#### Number of collisions

While not directly indicative of gait, collision frequency or obstacle contacts is a common metric in gait-related research involving obstacles. Seven studies reported collision frequency as a measure of mobility performance. Lombardi et al. (2018) found a significant correlation between visual field and mobility time, but not between visual field and mobility accidents. Other studies reported increased collisions among participants with PFL compared to age-matched healthy controls (Geruschat et al., 1998; Turano et al., 1999; Black et al., 1997), or those with mild PFL (Friedman et al., 2007) which were consistent with the negative effect of PFL on walking speeds. Conditions such as reduced lighting (Black et al., 1997; Geruschat et al., 1998; Bertaud et al., 2021) and distracting noise (Finger et al., 2016) exacerbated the risk of collisions for patients with PFL, suggesting that PFL increases the risk of collisions, particularly in challenging environments.

#### Spatiotemporal parameters

Studies utilizing sensors and cameras to measure gait revealed mixed results regarding the relationship between spatiotemporal parameters (Huang et al., 2022) and PFL. Some studies reported strong associations, with PFL associated with a broader base of support, shorter stride length, lower cadence, increased double support time, and greater variability in stride velocity and time (e.g., stride time, stance time, and swing time) (Bicket et al., 2020; Jian-Yu et al., 2021; Mihailovic et al., 2017; Odden et al., 2020). These gait alterations were further exacerbated under low illumination (whether simulated by wearing neutral density filters or actually reduced room lighting)(Bicket et al., 2020; Jian-Yu et al., 2021; Odden et al., 2020) and increased cognitive load (Mihailovic et al., 2017). When comparing individuals with a full visual field to those with PFL, studies have yielded inconsistent results. Study of Lee et al. (2021) found that the PFL group had significantly lower cadence, step length, stride length, single support time, and longer double support time, while other studies (De Alencar Gomes et al., 2018; Freitag et al., 2023) did not. Discrepancies may be attributed to the mild visual field loss in the latter studies.

In addition to spatiotemporal parameters, trunk parameters like body sway acceleration were investigated, with conflicting results. Studies of Ma et al. (2016a) and Ma et al. (2016b) indicated that patients with glaucoma experienced a larger range of trunk displacement, whereas Lee’s study found no significant effect of PFL on trunk sway while walking (Lee et al., 2021). Variability was also observed in foot parameters, including toe clearance, foot acceleration, crossing velocity during obstacle negotiation, and minimal vertical toe clearance (MTC) during normal walking. Participants with PFL showed higher toe clearance, foot acceleration, a larger coefficient of variation of MTC and lower crossing velocity, likely as a strategy to improve mobility safety (Ma et al., 2016a; Freitag et al., 2023). Overall, PFL appears to affect a range of gait parameters, with variability between studies possibly related to the extent of PFL, differing tasks and diverse assessment methods.

### Meta-analysis of the effect of peripheral visual field loss on gait parameters

#### Walking speed

**Figure 2:**
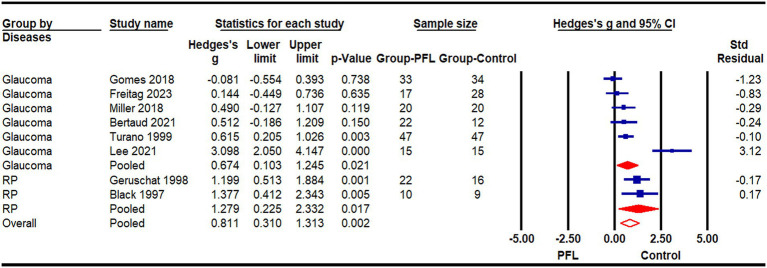
Meta-analysis of peripheral field loss (PFL) impact on walking speed with subgroup analyses for glaucoma and retinitis pigmentosa (RP).

**Figure 3:**
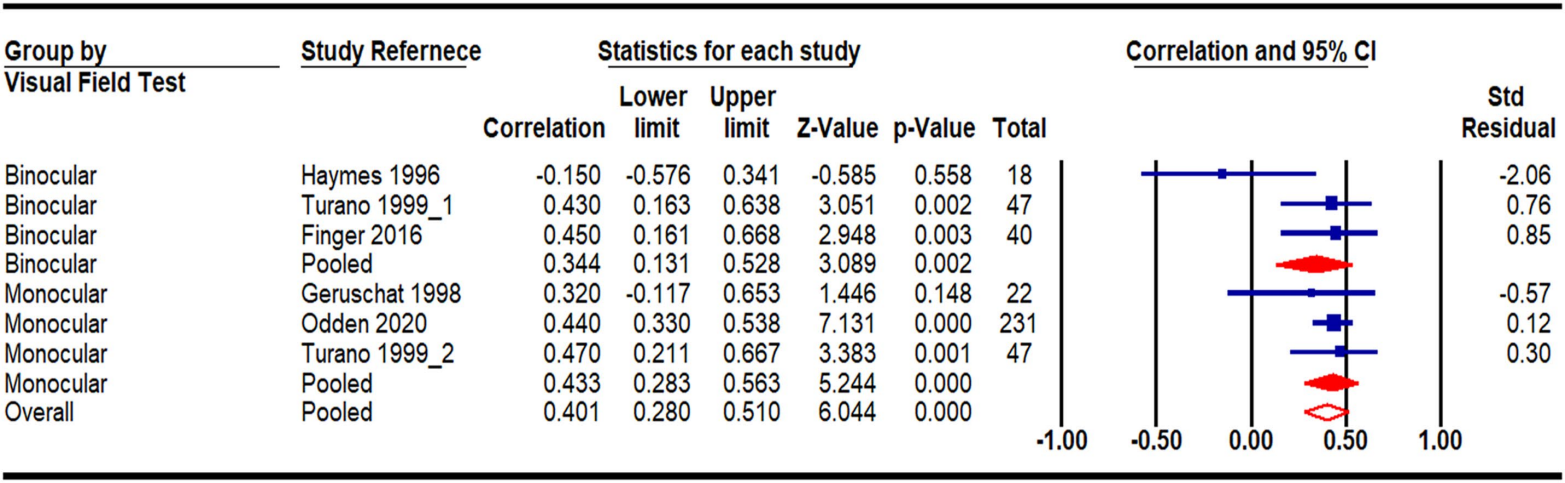
Meta-correlation analysis on the relationship between visual field and walking speed with subgroup analyses by types of visual field test (binocular vs. monocular).

A meta-analysis was conducted using data from eight studies (Geruschat et al., 1998; Turano et al., 1999; Miller et al., 2018; Lee et al., 2021; De Alencar Gomes et al., 2018; Bertaud et al., 2021; Black et al., 1997; Freitag et al., 2023) that compared the walking speeds of 186 patients with PFL to 181 age-matched individuals with normal vision (Figure 2). One study was excluded due to insufficient detail in its results (Ivanov et al., 2016). In studies reporting multiple outcomes, only the walking speed data was included. Consistent with the narrative review, the meta-analysis revealed a statistically significant impact of PFL on walking speed (Hedges’s g = 0.81, CI: 0.31 to 1.31, *p* < 0.05). The underlying cause of PFL, whether glaucoma or RP, did not significantly affect the degree of PFL impact on walking speed [χ^2^ (1) = 0.979, *p* = 0.32], implying a similar effect regardless of the cause. A meta-correlation analysis, pooled data from five studies (Haymes et al., 1996; Turano et al., 1999; Odden et al., 2020; Finger et al., 2016; Geruschat et al., 1998), further supported a significant relationship between the extent of PFL and walking speed (Figure 3). A smaller binocular visual field or a lower mean deviation in the monocular visual field was associated with slower walking speeds (r = 0.40, *p* < 0.001). The method of visual field testing was not associated with differences in walking speed outcomes [χ^2^ (1) = 0.513, *p* = 0.474].

#### Cadence

**Figure 4:**
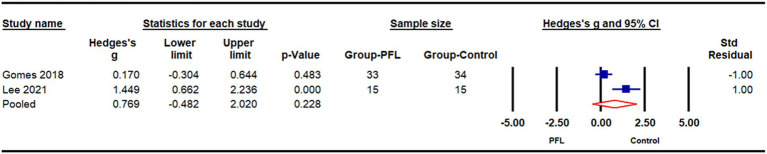
Meta-analysis of cadence differences between individuals with peripheral field loss (PFL) and normally sighted counterparts.

Two studies (Lee et al., 2021; De Alencar Gomes et al., 2018) comparing cadence differences between individuals with PFL (*n* = 48) and age-matched normally sighted counterparts (*n* = 49) were pooled in a meta-analysis (Figure 4). The result showed a biased-adjusted standardized mean difference (Hedges’s g) of 0.77 (95% CI: −0.48 to 2.02, *p* = 0.23), indicating no significant difference in cadence between the two groups.

## Discussion

This systematic review aimed to comprehensively evaluate the impacts of PFL on gait performance using various gait measures. Our findings indicate that under challenging conditions, such as dual-task scenarios or complex walking courses, patients with PFL experience further deterioration in gait performance. While variability across studies and often small sample sizes suggest caution in interpretation these results, the consistent findings across studies support the presence of real effects.

Our results found that PFL patients reduced their walking speed, especially in dim environments or when multitasking. The meta-analysis confirmed that people with PFL walked significantly slower than their normally sighted peers, with an average difference of 0.15 m/s (95% confidence interval: −0.03 to 0.33, Figure 2). Furthermore, the severity of visual field loss was significantly related to walking speed, with greater visual field loss correlating with slower speeds (Figure 3). This reduction in walking speed is likely influenced by the increased number of collisions experienced by PFL patients, which poses significant safety risks. To mitigate these risks, individuals with PFL often adopt compensatory gait strategies (Mihailovic et al., 2020; Wood et al., 2022; Ramulu et al., 2012).

Advanced technologies, such as sensors, video recording, and image analysis, have enabled the use of comprehensive spatiotemporal gait parameters, such as cadence, base of support. Additionally, variability in spatiotemporal gait parameters—which is often interpreted as a marker of impaired motor control and increased fall risk (Jungen et al., 2023)—has been shown to be significantly associated with PFL (Bicket et al., 2020; Jian-Yu et al., 2021; Mihailovic et al., 2017; Odden et al., 2020). In individuals with PFL, reduced peripheral vision may lead to cautious gait adaptations, resulting in inconsistent stride patterns as they compensate for impaired environmental perception. However, only a limited number of studies have investigated group differences in these metrics. Among the three studies that evaluated such differences, results were inconsistent. De Alencar Gomes et al. (2018) reported longer step lengths, increased swing times, and decreased double support times in glaucoma patients, while other studies (Lee et al., 2021; Freitag et al., 2023) reported increased stance and double support times, reduced step length and single support time in the PFL group. Yet the results from De Alencar Gomes et al. (2018) and Freitag et al. (2023) studies were not statistically significant. These discrepancies may be attributed to variations in participant age and walking test designs (single task vs. walking with obstacle or dual task). Given the variability in study designs and participant characteristics, only two studies on cadence were included in the meta-analysis, illustrating no significant difference between PFL and healthy controls (Figure 4). More research is warranted to examine the impact of PFL on specific spatiotemporal gait parameters.

This review also explored how challenging walking environments affect gait performance. Factors such as lighting conditions (Bertaud et al., 2021), walking course complexity (Finger et al., 2016), and dual-tasking (Gunn et al., 2019) were shown to influence gait patterns. For instance, glaucoma patients walked significantly slower in dim lighting compared to normal lighting (Bertaud et al., 2021). Several studies reported a more pronounced negative impact of PFL on gait during dual-tasking (Miller et al., 2018; Mihailovic et al., 2017; Freitag et al., 2023). However, results varied with some studies showing greater foot-placement errors during cognitive tasks (Miller et al., 2018; Mihailovic et al., 2017), while others found no adverse effects, potentially due to the mild visual field defects (Freitag et al., 2023). These findings underscore the intricate interplay between PFL severity, task demands, and gait alterations.

Despite these insights, we were unable to provide further clarity on how the extent of visual field loss impacts gait performance. Inconsistencies in visual tests used and parameters reported (e.g., mean deviation, area of the degree of the visual field) likely contribute to these challenges. Although this review did not identify studies examining the impact of specific regional PFL on gait, some evidence suggests that certain types of PFL, such as inferior hemifield visual field loss, may affect postural sway (Black et al., 2008). Participants with simulated lower or circumferential visual field defects demonstrated adaptive gait patterns, such as slower walking speed, greater stride length, and increased double support time (Neder and Cigali, 2022; Graci et al., 2009; Graci et al., 2010). Further research should focus on the effects of the extent and location of PFL on gait performance to design targeted interventions.

This systematic review is the first to comprehensively summarize gait measures from published studies and analyze the effect of PFL on gait performance without any restrictions on publication dates or study design. Despite providing valuable insights, the review has limitations, including variations in visual field assessments, potential sample bias from multiple papers by the same research group [six papers published by Ramulu’s group (Jian-Yu et al., 2021; Lee et al., 2021; Mihailovic et al., 2020; Mihailovic et al., 2017; Bicket et al., 2020; Odden et al., 2020)], and the lack of power analysis in most studies. Additionally, no study focused on kinematic gait parameters, such as trunk flexion and ankle plantar flexion, which could be affected by visual field loss due to balance deficits or increased foot probing the ground and warrant further investigation (Gazzellini et al., 2016; Hallemans et al., 2010).

## Conclusion

In conclusion, patients with peripheral field loss (PFL) frequently adapt their gait patterns to compensate for diminished visual input. Assessing these gait alterations is critical for evaluating fall risk and determining the efficacy of interventions in this population (Verghese et al., 2009; Ramulu et al., 2019). However, due to inconsistencies in visual field assessments across studies, this review could not quantify the severity of PFL or establish its generalized effects on gait. Future research should prioritize standardized methodologies to clarify how the severity and spatial distribution (e.g., superior, inferior, or hemianopia loss) of PFL influence functional mobility outcomes. Such efforts could inform targeted rehabilitation strategies and improve fall-risk stratification for this population.

## Data Availability

The raw data supporting the conclusions of this article will be made available by the authors, without undue reservation.
