# Supplementary material for: Effect of peripheral field loss on gait performance: a systematic review and meta-analysis
Source: Front Neurosci. 2025 Jun 13;19:1612793. doi: 10.3389/fnins.2025.1612793 (PMC12202335; doi:10.3389/fnins.2025.1612793)
Supplement: Supplementary file 1 [file Table_1.docx]

**SUPPLEMENTARY MATERIALS**

**Appendix 1: Literature search terms**

**Databases:** PubMed (Medline), Scopus, CINAHL, AMED Allied and Complementary Medicine (via Ovid), and the web of science

|  | **SEARCH THEMES** | |
| --- | --- | --- |
|  | ***Peripheral vision loss/ peripheral field loss*** | ***kinematic and spatiotemporal gait parameters*** |
| **SEARCH TERMS** | “Peripheral visual field loss” | “Gait” |
|  | “Peripheral vision loss” | “Gait parameters” |
|  | “Visual field damage” | “Gait analysis” |
|  | “Visual field defect” | “Gait patterns” |
|  | “Glaucoma” | “Gait dysfunction” |
|  | “Retinitis pigmentosa” | “Gait speed” |
|  | “RP” | “Gait time” |
|  | “Visual field loss” | “Walking” |
|  | “PFL” | “Mobility performance” |
|  |  | “Mobility” |
|  |  | “Gait characteristics” |
|  |  | “Kinematic parameters” |
|  |  | “Spatiotemporal parameters” |

**Appendix 2: CINAHL database search strategy**

| Search themes | Search terms |
| --- | --- |
| #1 (***Peripheral visual field loss)*** | Peripheral visual field loss OR Peripheral vision loss OR Visual field damage OR visual field defect OR Glaucoma OR Retinitis pigmentosa OR RP OR Visual field loss OR PVL |
| ***#2 (kinematic and spatiotemporal gait parameters)*** | Gait OR Gait parameters OR gait analysis OR gait patterns OR gait dysfunction OR gait speed OR gait time OR walking OR Mobility performance OR mobility OR gait characteristics OR kinematic parameters OR spatiotemporal parameters |
| #3 | #1AND#2 |

**Appendix 3: Reasons for the studies excluded from the meta-analysis**

| **Study reference** | **Types of PFL**  (real / simulated) | **Comparators**  (with/ without control group) | **Reason(s) for exclusion** |
| --- | --- | --- | --- |
| Bicket 2020 | Real PFL | No | - No control group - No correlation analysis for walking speed and visual field |
| Friedman 2007 | Real PFL | No | - No control group - No correlation analysis for walking speed and visual field |
| Gunn 2019 | Real PFL | No | - No control group - No correlation analysis for walking speed and visual field |
| Hall 2011 | Real PFL | No | - No control group - No correlation analysis for walking speed and visual field |
| Ivanov 2016 | Real PFL | Yes | - No detailed data |
| Jian-yu 2021 | Real PFL | No | - No control group - No correlation analysis for walking speed and visual field |
| Lombardi 2018 | Real PFL | No | - No control group - No correlation analysis for walking speed and visual field |
| Ma 2016 (a) | Real PFL | Yes | - No measure of walking speed or cadence |
| Ma 2016 (b) | Real PFL | Yes | - No measure of walking speed or cadence |
| Mihailovic 2017 | Real PFL | No | - No control group - No correlation analysis for walking speed and visual field |
| Mihailovic 2020 | Real PFL | No | - No control group - No correlation analysis for walking speed and visual field |
| Shakarchi 2019 | Real PFL | No | - No control group - No correlation analysis for walking speed and visual field |

PFL = peripheral field loss

**Appendix 4:**  **Characteristics of the included studies for narrative analysis**

| **Study reference** | **Study design** | **Participant characteristics** | **Vision assessments** | **Gait assessments** | | | **Results** |
| --- | --- | --- | --- | --- | --- | --- | --- |
|  |  | Population, sample size, age (y) & gender | VA (logMAR), CS & VF | Methods/ Tools | Measurement conditions | Parameters |  |
| Bertaud  (2021)  *France* | Cross-section | - Glaucoma   N=22  Age:56.4±10.1  F/M: 9/13   - Normal   N=12  Age:56.7±10.1  F/M: 5/7 | - VA   better eye  Glaucoma: 0.018±0.07  Normal: -0.03± 0.05  worse eye  Glaucoma: 0.19 ± 0.24  Normal: -0.04 ± 0.09   - CS   Glaucoma: -1.7± 0.2  Normal: 1.9±0.0   - VF:   HFA SITA standard 24-2  (IVF), Esterman  IVF:  Glaucoma: 32.3±22.3  Normal: -  Esterman score:  Glaucoma: 91.2± 27.3  Normal: 116.1±4.1 | Walked at their preferred walking speed following an established route with obstacles (8m) | 2 lighting conditions   - photopic lighting condition (P, 235 lux) - mesopic lighting condition with glare (M+G, 101 lux) | - PWS - PPWS - Mobility time - Mobility incidents | Effect of lighting:   - The mobility time was longer in both groups in M+G condition.   Effect of group:   - The number of mobility incidents was not significantly different for both groups. - PWS was slower in the glaucoma group in M+G compared with P.   Effect of lighting and group:   - Under M+G conditions, PWS and PPWS were significantly lower and mobility time was significantly longer for glaucoma group than for controls. |
| Bicket (2020)  *USA* | Cross-section | - Glaucoma   N= 213  Age:71.4±7.2 F/M: 100/113 | - VA   0.06 (0 to 0.14)   - CS   -   - VF:   HFA SITA standard 24-2  (MD)  Better-eye: −2.62 (−5.40 to −0.69)  Worse-eye: −5.72 (−13.35 to −2.64) | GAITRite Electronic Walkway (4.27 m)  Walked barefoot at a normal pace with habitual vision correction | 3 lighting conditions   - normal (1000 lux) - dim (2 lux) - normal post dim (NPD, return to 1000 lux) | - Spatiotemporal parameters included: velocity, cadence, base of support, stride length, % of time in double support, stride-to-stride variability, stride time, stride velocity, stance time, and swing time | - Lower IVF was significantly related to shorter strides, less cadence, increased double support time, broader base of support, and more variability in stride velocity, stance time, swing time, stride time, and stride length especially in the dim light. - For each 5 dB decrease in IVF, walking speed was significantly slower in both dim and NPD conditions. - In the transition from normal to dim lighting, reduced IVF sensitivity was associated with significant reduction of gait and cadence, shortening of stride length, lengthening of double support time, and increase in the variability of stride velocity, stride time, stance time, and swing time. |
| Black  (1997)  *Australia* | Cross-section | - RP   N=10  Age:45.2±11  F/M: 6/4   - Normal   N=9  Age:46.8±14  F/M: 7/2 | - VA   RP: 0.68±0.88  Normal: -0.15±0.08   - CS (Pelli-Robson chart)   RP: 1.05±0.70  Normal: 1.73±0.09   - VF:   HFA binocular 30-1 (average visual field extent, degree)  RP: 13.4±5  Normal: >30 | - Walked at normal walking speed for 20 m. (without cane) - Walked through an indoor pathway (1.2 m wide and 57.5 m long) which was divided into 3 rooms. | 2 lighting conditions   - low (26 lux, range 15-41) - high (447 lux, range 300-570)   Pathways   - Many obstacles (Rm 1 and Rm3) - Disability glare (Rm 2) | - PWS without obstacle - PPWS with obstacles avoidance - Total number of errors | - RP group’s mobility performance including safety (total number of errors) and PPWS was significantly worse than control group, especially under low illumination. |
| Finger (2016)  *Australia* | Cross-section | - Legal blindness (80% of patients were RP)   N=40  Age:53±16  F/M: 19/21 | - VA   2.3±1.0   - CS   -   - VF:   Goldmann kinetic perimetry (binocular, % of VF remained)  11.8±20.4 | Route travel tasks | 3 travel routes:   - 27m×2.6m straight path - 27m×2.6m, straight path with obstacles - 27m×2m with, obstacles, and background noise | - PWS - PPWS - Obstacle contacts | Effect of travel route：   - PPWS decreased, and body contacts increased as route and environmental complexity increased. - VF was correlated with PPWS in complex environment. |
| Freitag (2023)  Germany | Cross-section | - Glaucoma   N=19  Age:70.7±5.9  F/M:10/9   - Normal   N=30  Age: 70.9±5.1  F/M:17/13 | - VA   Glaucoma: -0.07±0.13  Normal: -0.1±0.08   - CS   -   - VF (MD, Median \| Range) ：   Glaucoma  OD: -0.67 \| 25.97  OS: -1.09 \| 22.00  Normal  OD: 0.59 \| 5.09  OS: 0.15 \| 5.92 | XSENS  MTw Awinda, Movella  Participants walk normally forth and back over a 10-m track for 180 seconds in each condition. | 4 tasks:   - Single walking task - Reaction time task (easy and difficult) - N-Back-Task   (easy and difficult)   - Letter fluency task   (easy and difficult) | - Stride length - Gait velocity - Minimum toe clearance (MTC) - Coefficient of variation (CoV) of stride length, gait velocity, and MTC - Dual-task cost (DTCs) for all parameters | Effect of Group   - The glaucoma group had significantly higher CoV of MTC compared with control group.   Effect of task   - CoV of stride length was higher in the single-task compared with each dual-task condition irrespective of group. - Results of DTC for CoV of stride length and gait velocity indicated better walking performance during the dual-task compared with the single-task. |
| Friedman (2007)  *USA* | Cross-section | - Bilateral glaucoma   N=74  Age:81.2±4.5  F/M: 43/31   - Unilateral glaucoma   N=76  Age:80.9±4.5  F/M: 37/39   - NO or possible glaucoma   N=1,064  Age:79.6±4.5  F/M: 649/415 | - VA   Bilateral glaucoma: 0.18 ± 0.35  Unilateral glaucoma: 0.11 ± 0.26  No glaucoma: 0.06 ± 0.22   - CS (Pelli-Robson chart)   Bilateral glaucoma: 1.46 ± 0.88 Unilateral glaucoma: 1.50 ± 0.70 No glaucoma: 1.52 ± 0.64   - VF   HFA SITA fast (MD)  Bilateral glaucoma:  -11.69 ± 8.38  Unilateral glaucoma:  -6.90 ± 6.04  No glaucoma:  -3.46 ± 5.11 | Walked quickly for 16.4 m (without cane) | - | - Walking speed - Number of collisions | - Bilateral glaucoma resulted in increased likelihood of bumping into objects and decreased speed (i.e., increased travel time). |
| Geruschat (1998)  *USA* | Cross-section | - RP   N=22  Age:44.4  F/M: /   - Normal   N=16  Age:38.2  F/M: / | - VA   RP: 0.26 (-0.16 to 1.66)  Normal: -0.11(-0.18 to 0.06)   - CS (Pelli-Robson chart)   RP: 1.34 (0 to 1.95)  Normal: 1.88 (1.4 to 2.1)   - VF   Goldmann perimeter  (Monocular, total area of functional retina in log unit)  RP:  OD: 2.29 mm^2^ (0 to 2.86 mm^2^)  OS: 2.27 mm^2^ (1.0 to 2.82 mm^2^)  Normal:  mean of 2.82 mm^2^ | Walked predefined courses quickly (without cane) | 2 mobility courses:   - Simple: 49m with obstacles - Complex: lived office corridors (444m) including obstacles and escalators   2 lighting conditions:   - Normal lighting with obstacles - Low lighting with obstacles (wearing goggles of neutral density filters that reduced transmission to 11%) | - Walking speed - Number of mobility incidents | Effect of group:   - RP subjects traveled slower than normally sighted subjects.   Effect of lighting:   - Both normally sighted and RP subjects traveled more slowly under reduced illumination.   Effect of lighting and group:   - RP subjects were five times more likely to have a mobility incident under reduced illumination than the normally sighted subjects. - Log CS and visual field extent, in combination, accounted for 69% of the variance in walking speed. |
| Gomes (2018)  *Brazil* | Cross-section | - Glaucoma   N=33  Age:68.4±8.0  F/M: 22/11   - Normal   N=34  Age:69.3±7.9  F/M:27/7 | - VA   Glaucoma: 0.07±0.18  Normal: 0.01±0.02   - CS   -   - VF   Octopus 1-2-3 (MD)  Glaucoma:  worse eye: -6.3±3.7  better eye: -4.8±1.8  Normal: - | GAITRite Electronic Walkway  Walked at a normal pace (5.74m) | - | - Spatiotemporal gait parameters included: velocity, cadence, step length, base of support, swing time, stance time and double support time | - No significant difference in gait parameters between glaucoma and control. |
| Gunn  (2019)  *Canada* | quasi- experimental design | - Glaucoma   N=13  Age:75.3±6.5 F/M: 5/8 | - VA   0.06±0.27   - CS (Melbourne Edge Test, LogCS)   1.68±0.37   - VF   HFA SITA standard 24-2 (IVF, MD)  better eye: -5.5 ± 4.7 dB  worse eye: -10.4 ± 7.7 dB | Optotrak Certus cameras with markers | 2 walking paths   - Precision walking: Walked across a 6 m path and stepped to the center of four sequential targets without stopping. - Obstacle negotiation: Walked across a 4.5m long path, trying to avoid poles.   3 tasks:   - No dual task - Counting dual task - Visual search task | - Gait speed | Effect of dual task:   - Participants walked slower in the counting and visual search dual task conditions than in the single task condition. |
| Hall  (2011)  *USA* | Cross-section | - RP or Usher's syndrome   N=10  Age: 51.0 (31-80)  F/M: 6/4 | - VA   0.47 (1.32 to 0.00)   - CS (Pelli Robson chart)   1.00 (0.10 to 1.65)   - VF   HFA 81-point full-field 3-zone screening, binocular  8°（2-18°） | Walked at a preferred and fast pace without an assistive device (6m) | - | - Walking speed - The number of falls in the previous year | - Preferred gait speed (median): 1.10 m/s - Fast gait speed (median): 1.68 m/s - Higher number of falls was related to faster gait speed. |
| Haymes (1996)  *Australia* | Cross-section | - RP   N=18  Age:44 (17-75)  F/M: 10/8 | - VA   0.0 to 1.60   - CS (Pelli-Robson)   0 to 1.8   - VF   Goldmann perimeter, (binocular kinetic visual, (% of total VF)  4% to 89% | Walked along different routes at a comfortable pace | 3 route designs:   - Route 1: quiet street with a concrete footpath (238m, flat, unobstructed) - Route 2:   Outdoor small business area with obstacles (pedestrians) (202m)   - Route 3: indoor shopping center with obstacles (254m) | PPWS | Effect of route design:   - A significant effect of route on PPWS, with decrease in mean PPWS with increasing route complexity. - Larger visual field correlated with higher PPWS for the more complex routes. |
| Ivanov (2016)  *Germany* | RCT | - RP   N=25  Age:51.2±11.2  F/M: /   - Normal   N=10  Age:35.5±10.4  F/M: / | - VA   RP: 0.3  Normal: -   - CS   -   - VF   Horizontal diameter of binocular VF  RP: 19.6°  Normal: - | Walked a course with or without obstacles (68m) | 2 lighting conditions:   - Normal (65 cd/m^2^) - Dim (5cd/m^2^) | PPWS | The average PPWS for the RP group was significantly slower than healthy control group. |
| Jian-yu (2021)  *USA* | Cross-section, longitudinal | - Glaucoma   N=241   - Age:   Normal/ mild VFL: 69.1±6.5 Moderate VFL: 72.3±8.7  Severe VFL: 70.1±7.1   - F/M:   Normal/ mild VFL: 59/60  Moderate VFL: 45/53-  Severe VFL: 12/12 | - VA   -   - CS   -   - VF   - | GAITRite Electronic Walkway  Walked a walkway back and forth twice at their normal walking speed. (4.88m) | 3 annual follow-ups | - Spatiotemporal gait parameters: stride velocity, cadence, base of support, stride length, stride-to-stride variability for velocity, base of support, stride length   (All gait metrics were converted to z-score units) | - At baseline, people with more severe visual field damage reported an increase in base of support, variability of stride length and stride velocity, and decrease in stride length and stride velocity. - Within each glaucoma severity group, stride length declined over time. Patients with more severe VF damage levels had further reductions in stride velocity and cadence. |
| Lee  (2021)  *Korea* | Cross-section | - Glaucoma   N=15  Age:72.87±3.38  F/M:11 /4   - Normal   N=15  Age: 72.73±3.88  F/M: 11/4 | - VA - CS   -   - VF   - | Tekscan F-scan system  Walked a straight path with and without obstacle at a normal pace (6m) | - | - Spatiotemporal: gait speed, cadence, gait cycle, stance time, center of force deviation, center of force excursion | - Compared with the healthy control group, patients with glaucoma had significantly slower gait speed, cadence, and longer gait cycle time and stance time in both with and without obstacle conditions. |
| Lombardi (2018)  *France* | Cross-section | - Glaucoma   N=32  Age: 59.31±12.42  F/M: 16/16 | - VA   Better eye: 0.0175±0.047  Worse eye: 0.0362±0.055   - CS   1.79±0.14   - VF   Esterman  106.6±21.52 | Walked an established route within an indoor office with obstacles at a preferred walking speed (15m) | - | - mobility time - mobility incidents | - The Esterman score significantly correlated with mobility time. - VF was not found related to mobility incidence. |
| Ma  (2016) (a)  *USA* | Cross-section | - Glaucoma   N=9  Age:63.7±8.57  F/M: 4/5   - Normal   N=10  Age: 60.7±4.99  F/M: 3/7 | - VA   -   - CS   -   - VF   - | Shoe-based wearable sensor platform   - 10-Meter-Walk test - Obstacle course (10m with obstacles) | - | - Anterior-posterior foot acceleration: Maximum, minimum, median, mean, range, amplitude. - Medio-lateral foot acceleration: Maximum, minimum, median, amplitude - Vertical foot acceleration: Maximum, median, root-mean-square | Glaucoma patients had higher mean and median foot acceleration along the anterior-posterior direction, but lower foot acceleration along the medio-lateral direction. |
| Ma  (2016) (b)  *USA* | Cross-section | - Glaucoma   N=9  Age:63.7±8.57  F/M: 4/5   - Normal   N=10  Age: 60.7±4.99  F/M: 3/7 | - VA   -   - CS   -   - VF   - | Shoe-based wearable sensor platform   - 10-Meter-Walk test - Obstacle course (10m with obstacles) | - | - Spatiotemporal: Stride time, cadence, displacement in medio-lateral and anterior-posterior directions,   sway speed in medial-lateral and anterior-posterior directions, force (force generated by contacting the ground) | - 10-meter walking test:   Range of displacement amplitude along ML direction was much larger in glaucoma patients.  The median value along AP direction was significantly smaller in glaucoma patients.   - Obstacle course:   Glaucoma patients had smaller minimum foot acceleration but larger median foot acceleration along ML direction over gait cycles.  The median value of foot acceleration along AP direction was smaller in glaucoma patients. |
| Mihailovic (2017)  *USA* | Cross-section | - Glaucoma   N=239  Age:70.6±7.6  F/M: 117/122 | - VA   0.06 (-0.01 to 0.16)   - CS (MARS chart)   1.72 (1.64 to1.76)   - VF   HFA standard 24-2 MD)  Better-eye: -2.56 (-5.41, -0.68)  Worse eye: -5.33 (-12.39, -2.64) | GAITRite Electronic Walkway  Walked at normal speed without obstacle (4.88m) | 3 tasks:   - No dual task - Carry a cup - Carry a tray | - Spatiotemporal:   drift, step length, base of support, stride length, stride velocity, gait speed stride-to-stride variability for Step length, base of support, stride length, stride velocity, gait speed | Effect of task:   - No dual task:   Worse IVF sensitivity was associated with a broader base of support, greater coefficient of variation in step length, stride length and stride velocity.   - Dual task:   Worse IVF sensitivity was associated with a broader base of support, shorter step or stride length, slower stride velocity, greater drift, greater variability in step length, stride length, and stride velocity.   - Walking speed was 2.76-cm/s slower for every 5-dB decrement in the IVF sensitivity during the tray carrying condition. |
| Mihailovic  (2020)  *USA* | Cross-section | - Glaucoma   N=239  Age:70.5±7.6  F/M: 115/124 | - VA   0.06 (-0.02 to 0.16)   - CS (MARS chart)   1.72 (1.64 to 1.76)   - VF   HFA standard 24-2 (MD)  better-eye: -2.6 (-5.4, -0.7)  worse eye: -5.7 (-12.9, -2.8) | GAITRite Electronic Walkway  Walked at normal speed without obstacle (4.88m) | - | - Spatiotemporal:   velocity, cadence, base of support, stride length, and percent of the stride time in double support,  coefficients of variation for stride length, stride velocity, stride time, stance time, and swing time   - Fall data collection - Physical activity | - Increased velocity, cadence, and stride length were associated with more steps per day, whereas a greater percent of the cycle time spent in double support and greater variability in gait were associated with fewer steps per day. - Higher gait velocity and faster cadence were associated with fewer falls/step. - A larger percentage of time spent in double support, and greater variability in swing time were associated with more falls/steps. |
| Miller (2018)  *Canada* | Cross-section | - Glaucoma   N=20  Age74.3±6.3  F/M: 14/6   - Normal   N=20  Age:70.7±6.8  F/M: 14/6 | - VA   -   - CS   -   - VF   HFA standard 30-2 (better eye, MD)  -8.88±5.57 (glaucoma)  1.10±1.70 (normal) | Optotrak Certus motion capture camera  Walked at normal speed and stepped to the center of a series of four sequential targets | 3 conditions:   - No dual task - Counting backward - Visual Searching | - Foot -placement error - Foot-placement error variability - Gait speed | Effect of task:   - Subjects walked slower in the count dual task condition compared with the visual search dual task condition, and fastest in the single task condition.   Effect of group and task:   - Glaucoma demonstrated greater foot-placement error and foot-placement error variability than controls only in the count dual task. - Greater field loss was associated with greater foot-placement error and foot-placement error variability in dual task conditions. - Glaucoma walked slower than normal but not significantly different. |
| Odden (2020)  *USA* | Cross-section | - Glaucoma   N=231  Age (IQR):70.6 (54,75)  F/M:114/117 | - VA   -   - CS   -   - VF   HFA standard 24-2, suprathreshold peripheral 60 screening (IVF)  Abnormal central IVF points (%): 1.92 (0,13.46)  Abnormal peripheral IVF points (%): 5.36 (1.79,17.63) | GAITRite Electronic Walkway  Walked at normal speed without obstacle (4.88m) | - | - Spatiotemporal: base of support, step length, stride length, stride velocity, walking speed, variability in step length, variability in stride length, and variability in stride velocity | - Greater visual field damage was significantly associated with shorter steps, broader base of support, greater variability in step length, stride length, and stride velocity |
| Shakarchi  (2019)  *USA* | Cross-section | - Glaucoma   N=151  Age: 70±6.8y  F/M: 79/72 | - VA   0.92±1.4   - CS   -   - VF   HFA SITA standard 24-2  -5.5±0.8 | GAITRite Electronic Walkway  Walked at usual-pace walking (4.88m) | - | - Spatiotemporal:   gait speed, stride length, stride velocity, and base of support | - Glaucomatous IVF contributed to reduced function in all outcomes, but it explained only 15% to 35% of functional impairment (35% for base of support). |
| Turano (1999)  *USA* | Cross-section | - Glaucoma   N=47  Age:65.1  F/M:/   - Normal   N=47  Age:60.2  F/M:/ | - VA   Glaucoma: 0.15±0.36  Normal: 0.08±0.10   - CS (Pelli-Robson chart)   Glaucoma: 1.49±0.25  Normal: 1.80±0.11   - VF   HFA SITA 24-2 (MD)  Glaucoma:  better eye: -10.1±8.41  worse eye: -16.5±10.00  Normal: - | Walked 2 courses at a self-selected speed. | 2 course conditions:   - Course 1: 29m, no obstacle - Course 2: 29m, obstacles, turns | - Walking speed - Number of mobility incidents | Effect of group:   - Glaucoma group walked significantly slower than the normal-vision group. - The number of mobility incidents (collisions, stumbles, or orientation problems) was almost twice as high in the glaucoma group than in the normal–vision group. - Walking speed in glaucoma subjects was highly correlated with visual field defects.   Effect of course:   - Greater path complexity slowed walking speed in both groups. |
